# The Effects of Positioning During Colonoscopy on Efficacy and Post-procedure Comfort

**DOI:** 10.5152/tjg.2025.24439

**Published:** 2025-01-06

**Authors:** Mustafa Ergin, Gülencan Yumuşak Ergin, Fatih Kıvrakoğlu, Mehmet İbiş

**Affiliations:** 1 Department of Gastroenterology, Aksaray University Faculty of Medicine, Aksaray Training and Research Hospital, Aksaray, Türkiye; 2 Department of Anesthesiology and Reanimation, Aksaray University Faculty of Medicine, Aksaray Training and Research Hospital, Aksaray, Türkiye; 3 Department of Gastroenterology, Osmaniye State Hospital, Osmaniye, Türkiye; 4 Department of Gastroenterology, Ufuk University Faculty of Medicine, Dr. Rıdvan Ege Hospital, Ankara, Türkiye

**Keywords:** Colonoscopy, left lateral, right lateral, postprocedural discomfort, visual analog scale

## Abstract

**Background/Aims:**

There are studies with different results on improving effectiveness and patient comfort by increasing lumen distention through positioning during colonoscopy. In our study, we aimed to compare colonoscopy outcomes and post-procedural patient comfort in the left-lateral and right-lateral positions.

**Materials and Methods:**

A total of 231 patients who underwent screening colonoscopy were included. Patients were randomized to either the left-lateral or the right-lateral position. Patient age, sex, comorbidities, body mass index (BMI) values, times to reach the cecum, ileum intubation rates, total procedure times, and colonoscopy findings were compared. Pain and discomfort after the procedure were evaluated and compared with the visual analog scale (VAS) scores at 30 minutes, 6 hours, and 24 hours.

**Results:**

Colonoscopy was performed in the left-lateral position in 129 patients and in the right-lateral position in 102. The distributions of age, sex, comorbidities, and BMI values were similar in both groups. The time to reach the cecum and the total procedure time were similar in both groups. There were no significant differences in the findings detected by colonoscopy. There was no significant difference in the post-procedural VAS scores.

**Conclusion:**

This study failed to show a difference in colonoscopy outcomes and postprocedural discomfort between the left- and right lateral positions.

**Main Points**

- There is no difference in terms of the time to reach the cecum and the total procedure time in colonoscopy procedures performed in the left and right lateral positions.
- Whether colonoscopy is performed in the left or right lateral position does not affect the polyp detection rate or procedure efficiency.
- There is no difference in the post-procedure visual analog scale (VAS) scores for both positions.
- Adequate bowel cleansing and sufficient time for the procedure are essential for a screening colonoscopy.

## Introduction

Colorectal cancer ranks third among all cancers in terms of incidence and second in terms of mortality.^1^ The regions with the highest incidences of colon cancer are Europe, Australia/New Zealand, and North America. It ranks first among men in Hungary and women in Norway. The incidence of rectal cancer is highest in East Asia.^2^

Most colorectal cancers occur through the adenoma-carcinoma sequence. Approximately 70% of sporadic colorectal cancers develop from adenomatous polyps. Colorectal cancer screening is aimed at the detection and early treatment of adenomas and early-stage cancers. Colonoscopy, sigmoidoscopy, computed tomography (CT) colonography, and stool-based tests can be used for this purpose. Colonoscopy is the ideal method for detecting adenomas. Simultaneously, endoscopic removal of polyps detected during colonoscopy by polypectomy reduces the incidence and mortality of colorectal cancer.^3^ Colorectal cancer screening is recommended starting at the age of 45 years.^3^

The left lateral decubitus position is commonly used as the traditional starting position in colonoscopy. In addition, the procedure can be performed in different positions, such as right lateral, supine, and prone. To increase the effectiveness and success of the colonoscopy procedure and patient comfort both during and after the procedure, there are some studies in the literature on different starting positions as an alternative to the preferred left lateral decubitus position for the colonoscopy procedure, but they are few in number.^4,5^ Position change or patient rotation is based on the knowledge that increasing luminal distention may improve mucosal visualization. Considering that the air in the lumen is elevated, the right colon is most insufflated when the patient is in the left lateral decubitus position, the transverse colon is most insufflated in the supine position, and the left colon is best insufflated and visualized in the right lateral position.^4,6^ Anatomically, the left colon, often the sigmoid colon, is generally the most difficult to visualize and navigate during colonoscopy. Moreover, starting the procedure in the left lateral position allows air to escape from the left colon more easily, the lumen collapses, and the procedure becomes more difficult. To perform the procedure more easily and increase its effectiveness, it is important to visualize the sigmoid colon easily and pass it with the colonoscope without looping.^4^ Therefore, it may be reasonable to start the colonoscopy procedure in the right lateral position.

Approximately one-third of patients undergoing colonoscopy experience abdominal pain, nausea, or bloating, which can last hours to several days. Fortunately, serious complications, such as bleeding, perforation, and death, are rare, with an overall incidence of 0.28%.^7,8^

Abdominal pain, abdominal discomfort, and bloating after colonoscopy are less severe, but more common adverse effects that may affect patient compliance with future colonoscopies.^9^ The most commonly reported minor adverse effects of colonoscopy are bloating (2.6-25%) and abdominal pain or discomfort (2.5-11%). Abdominal discomfort may be caused by colon spasm, gas distension, or mechanical or barotrauma.^9^ Techniques such as avoiding and reducing the looping of the colonoscope and minimizing air insufflation help reduce these symptoms.^10^ Post-colonoscopy abdominal discomfort caused by gas bloating is usually self-limiting and rarely requires hospitalization.

This study aimed to determine how performing colonoscopy in the right lateral decubitus position affects the success, effectiveness, and patient comfort after the procedure compared to the standard left lateral decubitus position.

## MATERIALS AND METHODS

### Patient Selection and Study Design

**Figure 1.**
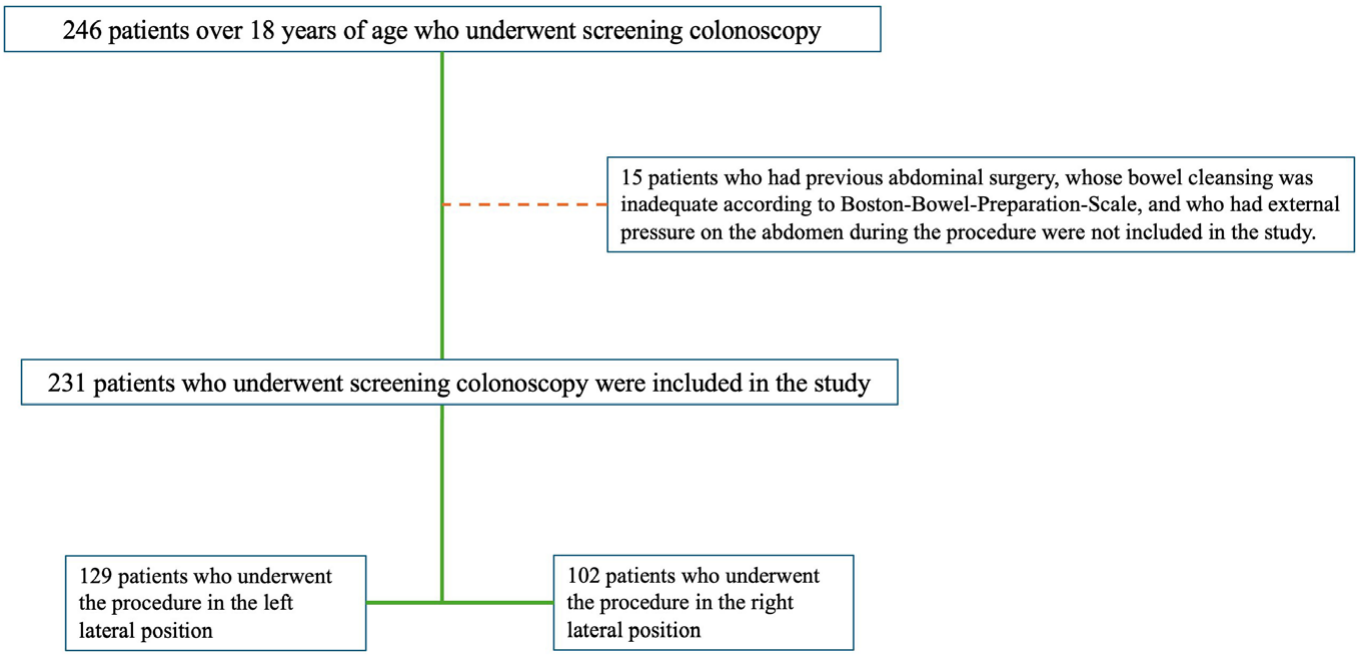
Study design.

Among 1557 patients aged >18 years who underwent colonoscopy in Aksaray University Training and Research Hospital between February 1, 2022, and November 30, 2023, 231 patients who underwent screening colonoscopy were included in the study (Figure 1).

The demographic characteristics and colonoscopy reports of these patients were retrospectively examined using a hospital information system. Age, sex, comorbidities, medications used, body mass index (BMI) values, colonoscopy indications, medications used for bowel cleansing, medications used for sedation, and the position in which the procedure was performed were recorded for patients who underwent colonoscopy. Patients who had previous abdominal surgery, inadequate bowel cleansing according to the Boston Bowel Preparation Scale (BPPS),^11^ or external pressure on the abdomen during the procedure were not included in the study (Figure 1). The study received approval from the Aksaray University Faculty of Medicine Clinical Research Ethics Committee (number: 2023/22-04, date: 23.11.2023). Informed consent was obtained from all participants in accordance with the principles of the Declaration of Helsinki.

The procedures were performed by a single experienced endoscopist (who had performed more than 5000 colonoscopies). All patients were given an appropriate diet and laxative medications before the procedure. All procedures were performed using the same device (Olympus Evis Exera CV-190). Fluoroscopy was not performed. Patients were divided into 2 groups: left lateral decubitus and right lateral decubitus. The left lateral and right lateral decubitus positions were chosen randomly before the procedure. No positional changes were made to the patients during the procedure. During the procedure, all patients were anesthetized by an anesthesiologist with intravenous sedation and propofol, according to the standard protocol. Opioids and other analgesics were not administered to the patients. As carbon dioxide insufflation was not possible in our unit, insufflation was performed with room air in all patients. Information on whether cecal intubation was performed, cecum access time, exit time, and whether ileum intubation was performed were recorded from the patients’ procedure reports. Polyps detected during the procedure, their location, and other pathological findings were recorded.

Abdominal pain/discomfort conditions of patients who were routinely evaluated for complications after the procedure were questioned and recorded according to the visual analog scale (VAS).^12^ Visual analog scale scores range from 0 to 10; with higher scores indicating more pain and a score of 0 indicating no pain. Visual analog scale scores of 0 were considered to indicate no pain, 1-3 to indicate mild pain, 4-6 to moderate, and 7-10 to indicate severe pain, respectively. Patients whose VAS scores were not 0 before the procedure were excluded from the study. Visual analog scale scores were evaluated by an experienced nurse 30 minutes, 6 hours, and 24 hours after the procedure.

### Statistical Analysis

Statistical package for social sciences (SPSS) version 29.0 (IBM SPSS Corp.; Armonk, NY, USA) was used to analyze the data. Descriptive statistics are shown as numbers (n) and percentages (%) of the qualitative data. For quantitative data, median and minimum-maximum values were given because normal distribution assumptions were not met. Comparison of categorical variables between groups was performed using Pearson’s chi-square or Fisher’s exact test. When comparing continuous variables in 2 independent groups, the Mann–Whitney *U* test was used because the assumption of normal distribution was not met. For all statistics, the type 1 margin of error (alpha) was accepted as 0.05.

## Results

A total of 231 patients who underwent screening colonoscopy were included in the study. One hundred six of these patients were female, and 125 were male. The demographic characteristics of the patients are shown in Table 1.

The cecum was intubated in 227 of 231 patients, and the ileum was intubated in 216 patients. The ileum was normal in 214 patients who were intubated. Colonoscopy findings were normal in 140 of 231 patients, polyps in 70 patients, diverticula in 13 patients, malignancy in 7 patients, and inflammatory bowel disease in 1 patient. Relevant findings according to colon location are shown in Table 2.

The procedure was performed in the left lateral position in 129 of 231 patients and in the right lateral position in 102 patients. The age and sex distributions were similar in both groups (Table 3). There were no significant differences between the groups in terms of BMI values or comorbidities. The cecal and ileal intubation rates were similar between the groups. The findings for the left and right-lateral positions are compared in Table 3.

Among 231 patients whose VAS scores were evaluated at the 30th minute, 6th hour, and 24th hour, 212 patients had no pain or discomfort at the 30th minute, 18 patients had mild pain or discomfort, and 1 patient had moderate pain or discomfort. None of the patients showed any severe symptoms. The current and other findings are presented in Table 4. The findings comparing VAS scores for the left and right lateral decubitus positions are shown in Table 5.

## Discussion

The detection and removal of polyps during screening colonoscopy are essential for the effective prevention of colon cancer. Colonoscopic examination remains the gold standard for polyp detection and treatment.

Traditionally, a colonoscopy is performed with the patient in the left lateral position.^13^ However, there is no evidence to support the effectiveness or advantages of this starting position.^14^ In addition, the procedure can be performed on patients in positions such as the right lateral, prone, and supine positions, or the position can be changed during the procedure. We performed the procedures in the left and right lateral positions and compared the findings.

By changing the patient’s position, the colon moves within the abdomen, and fluid and gas move within the lumen. For years, radiologists have used these changes to optimize images during barium examinations. It has been suggested that adjusting the patient’s position to bring the colon segments to their highest point in the abdomen improves luminal distension and, therefore, lesion detection during colonoscope withdrawal.^15^ However, endoscopists’ practices vary. Some endoscopists examine the colon with the patient in a single fixed position, while others routinely change positions during colonoscopy withdrawal.^16^ These differences may be related to difficulties in moving patients or uncertainty regarding the benefits of position change. In our study, patients who underwent the procedure in the left or right position were examined in a fixed position, and no positional changes were made.

Various studies have shown that examining the transverse colon with the patient in a supine position increases polyp detection.^17-19^ In addition, in the study conducted by Shah et al^20^, adenoma detection rates were found to be similar in the prone and lateral positions, and their relationship with the position was not shown. In another study, it was observed that dynamic position change did not affect the procedure time, and polyp detection rates were similar in different positions; however, in subgroup analyses, it was emphasized that dynamic position change for the transverse colon significantly increased the adenoma and polyp detection rates.^21^ In the study in which we compared the left and right lateral positions, no significant difference was observed between polyp detection rates and other pathological findings in both positions. In the subgroup analyses, when all colon segments were evaluated separately, the findings were similar. Although various studies have reported the advantages and disadvantages of positions compared to each other, we believe that other factors are more important than position in polyp detection. We believe that all segments should be adequately evaluated by ensuring adequate bowel cleansing and distension in the lumen and that the procedure should be performed in sufficient time for examination.

Studies have also shown that patient position during colonoscopy affects the time taken to reach the cecum. In 1 study, the time to reach the cecum was significantly lower in the prone position than in the supine position, and there was less need for abdominal compression.^22^ The review, which included 10 randomized controlled trials and 2083 patients and presented data on the effectiveness and safety of the starting position during colonoscopy, showed that all starting positions reduced the mean access time to the cecum for colonoscopy compared with the left lateral position. However, because the certainty of the evidence is very low, these data should be interpreted with caution.^14^ Another meta-analysis showed that the starting position of colonoscopy did not affect the time taken to reach the cecum.^23^ In the ROLCOL (Right Or Left in COLonoscopy) study, the cecal intubation time in the right lateral position was found to be significantly shorter than that in the left lateral position.^24^ In our study, the time to reach the cecum was similar for the left lateral and right lateral positions. We believe that endoscopists’ experience is important in this regard. In our study, the procedures were performed by a single experienced endoscopist, and the endoscopist’s previous experience with the procedure in the right lateral position may have affected this situation. However, although the procedure time was not affected, the left lateral position may be more advantageous than the right lateral position, especially in terms of evaluating and locating the anus at the beginning of the procedure, which may provide greater comfort for the endoscopist. In a recent study designed by Landry et al^25^, the effect of performing the procedure in the left lateral and right lateral positions on the endoscopist’s risk of musculoskeletal injury was observed. Although there was a risk of musculoskeletal injury in both groups, this risk was higher in the right lateral position. This situation is also important when deciding which position the procedure will be performed in. In fact, in the relevant study, endoscopists preferred the left lateral position as more comfortable. It may be difficult for the endoscopist to lie down over the patient when he/she is in the right-lateral position.

Although colonoscopy procedures are invasive, they are often performed under conscious sedation or in awake patients and are generally well tolerated. The risk of adverse events is low. Up to 10% of patients may experience moderate-to-severe discomfort during colonoscopy, including abdominal pain, cramping, nausea, and bloating.^26^ No serious adverse events were observed in either the left or right lateral positions in the patients included in our study. Symptoms such as bloating, abdominal pain, and changes in bowel function may persist after colonoscopy in up to 34% of the patients, and the majority (94%) return to normal within 2 days or less.^27^ This feeling of discomfort experienced by patients during and after the procedure may affect their functional status and approach to possible subsequent procedures. Therefore, it is important to inform patients about the discomfort that they may experience. Appropriate conditions should be established for easy access and application when complications develop.

Various precautions should be taken to avoid pain and discomfort after the procedure. During colonoscopy, insufflation of carbon dioxide into the lumen instead of room air may reduce pain.^10^ In another study, propofol sedation, higher case volume by endoscopists, the use of new endoscopes, and adequate bowel cleansing were significantly associated with a lower likelihood of painful colonoscopy. Pain scores after colonoscopy were similar.^28^ In a study by Cankurtaran et al^29^, it was shown that thermal therapy with an external hot pack applied to the abdomen of patients reduced post-colonoscopy pain.

There is limited information in the literature about whether the position in which the procedure is performed is important to avoid pain and discomfort after colonoscopy, and conflicting results have been reported. Zhao et al^30^ stated that pain scores decreased in the supine position compared to the left lateral position. In the ROLCOL study, patients found the procedure more comfortable and felt less pain and discomfort in those who started the procedure in the right lateral position than in those who started in the left lateral position. This difference was more pronounced in women and in those with a history of abdominal surgery.^23^ Similar pain scores were reported in the left and right lateral positions in a study by Bayupurnama et al^31^ Other studies in the literature reported no significant difference in pain scores in the left lateral and right lateral positions.^4,32^ In our study, patients who underwent the procedure in the left and right lateral positions were questioned about discomfort and pain after the procedure according to the VAS score. None of the patients in either group had any severe symptoms. No significant differences were found between the left and right lateral positions. Compared with previous studies, we conducted a study with the highest number of patients.

Our study aimed to compare the left and right lateral positions. Designing the study with a larger number of patients and adding supine and prone positions would have enabled us to provide more comprehensive comments. The limiting features of the study were that the VAS score used to evaluate pain and discomfort provided subjective data and that there were differences in pain threshold and perception for each patient.

In conclusion, colonoscopy is an important modality used for screening in the detection of malignant and premalignant lesions, and performing the procedure in the left or right lateral position has no advantage over the other.

## Figures and Tables

**Table 1.** Demographic Characteristics of the Patients

Age (years) (median) (min-max)	56.8 (23-78)
Sex	n (%)
Female	106 (45.9)
Male	125 (54.1)
Comorbidities	n (%)
Diabetes mellitus	61 (26.5)
Hypertension	64 (27.7)
Chronic obstructive pulmonary disease and asthma	15 (6.5)
Cardiovascular disease	22 (9.5)
Neuropsychiatric disease	1 (0.4)
None	68 (29.4)
BMI	n (%)
< 18.5	5 (2.2)
18.5-24.9	123 (53.2)
25-29.9	90 (39)
> 30	13 (5.6)

BMI, body mass index

**Table 2.** Colonoscopy Findings of the Patients

Colonoscopic Diagnosis	n (%)
Normal	140 (60.6)
Polyp	70 (30.3)
Cancer	7 (3)
Diverticulum	13 (5.7)
Others	1 (0.4)
Time to reach the cecum (median) (minutes) (min-max)	2 (1-10)
Withdrawal time (median) (minutes) (min-max)	11 (8-32)
Total colonoscopy procedure time (median) (minutes) (min-max)	14 (9-35)
Cecum intubation	
Yes	227 (98.2)
No	4 (1.8)
Ileum intubation	
Yes	216 (93.5)
No	15 (6.5)
Ileum	
Not intubated	15 (6.5)
Normal	214 (92.6)
Ulcer	2 (0.9)
Cecum	
Not intubated	4 (0.4)
Normal	221 (97)
Polyp	4 (1.7)
Cancer	–
Diverticulum	2 (0.9)
Others	–
Ascending colon	
Normal	210 (90.9)
Polyp	10 (4.3)
Cancer	2 (0.9)
Diverticulum	8 (3.5)
Others	1 (0.4)
Transverse colon	
Normal	198 (85.7)
Polyp	24 (10.4)
Cancer	1 (0.4)
Diverticulum	8 (3.5)
Others	–
Descending colon	
Normal	198 (85.7)
Polyp	20 (8.7)
Cancer	–
Diverticulum	12 (5.2)
Others	1 (0.4)
Sigmoid colon	
Normal	174 (75.3)
Polyp	32 (13.9)
Cancer	4 (1.7)
Diverticulum	21 (9.1)
Others	–
Rectum	
Normal	210 (90.9)
Polyp	21 (9.1)
Cancer	–
Diverticulum	–
Others	–
Anal canal	
Normal	209 (90.5)
Hemorrhoids	14 (6.1)
Fissure	1 (0.4)
Hypertrophic anal papilla	7 (3)

**Table 3.** Comparison of Left Lateral and Right Lateral Positions

	Left Lateral	Right Lateral	*P*
Age (years) (median) (min-max)	58 (23-78)	55.5 (27-75)	.57^a^
Sex n (%)^b^			.63^c^
Female	61 (47.3)	45 (44.1)	
Male	68 (52.7)	57 (55.9)	
Total	129 (100)	102 (100)	
Comorbidities n (%)^b^			
Diabetes mellitus	31 (24.2)	30 (29.4)	.37^c^
Hypertension	34 (26.6)	30 (29.4)	.63^c^
Chronic obstructive pulmonary disease and asthma	10 (7.8)	5 (4.9)	.37^c^
Cardiovascular disease	11 (8.6)	11 (10.8)	.57^c^
Neuropsychiatric disease	1 (0.8)	–	1.00^d^
None	37 (28.9)	31 (30.4)	.80^c^
BMI n (%)^b^			.55^d^
< 18.5	4 (3.1)	1 (1)	
18.5-24.9	68 (53.1)	55 (53.9)	
25-29.9	51 (39.8)	39 (38.2)	
> 30	6 (4)	7 (6.9)	
Total	129 (100)	102 (100)	
Number of patients ileum intubated n (%)^b^	120 (93)	96 (94.1)	.48^d^
Number of patients cecum intubated n (%)^b^	126 (97.7)	101 (99)	.63^d^
Ascending colon n (%)^b^			.13^d^
Normal	120 (93)	90 (88.2)	
Polyp	4 (3.1)	6 (5.9)	
Cancer	2 (1.6)	–	
Diverticulum	2 (1.6)	6 (5.9)	
Others	1 (0.7)	–	
Total	129 (100)	102 (100)	
Transverse colon n (%)^b^			.057^d^
Normal	116 (89.9)	82 (80.4)	
Polyp	8 (6.2)	16 (15.7)	
Cancer	–	1 (1)	
Diverticulum	5 (3.9)	3 (2.9)	
Total	129 (100)	102 (100)	
Descending colon n (%)^b^			.08^d^
Normal	112 (86.8)	86 (84.3)	
Polyp	7 (5.4)	13 (12.8)	
Cancer	–	–	
Diverticulum	9 (7)	3 (2.9)	
Others	1 (0.8)	–	
Total	129 (100)	102 (100)	
Sigmoid colon n (%)^b^			.88^d^
Normal	98 (76)	76 (74.5)	
Polyp	17 (13.2)	15 (14.7)	
Cancer	3 (2.3)	1 (1)	
Diverticulum	11 (8.5)	10 (9.8)	
Total	129 (100)	102 (100)	
Rectum n (%)^b^			.73^d^
Normal	118 (91.5)	92 (90.2)	
Polyp	11 (8.5)	10 (9.8)	
Total	129 (100)	102 (100)	
Anal canal n (%)^b^			.09^d^
Normal	121 (93.8)	88 (86.3)	
Hemorrhoids	4 (3.1)	10 (9.8)	
Fissure	–	1 (1)	
Hypertrophic anal papilla	4 (3.1)	3 (2.9)	
Total	129 (100)	102 (100)	
Colonoscopic diagnosis n (%)^b^			.29^d^
Normal	81 (62.7)	59 (57.8)	
Polyp	33 (25.6)	37 (36.3)	
Diverticulum	9 (7)	4 (3.9)	
Cancer	5 (3.9)	2 (2.0)	
Others	1 (0.8)	–	
Total	129 (100)	102 (100)	
Time to reach the cecum (median) (minutes) (min-max)	2 (1-8)	2 (1-10)	.62^a^
Withdrawal time (median) (minutes) (min-max)	11 (8-25)	11 (8-32)	.59^a^
Total colonoscopy procedure time (median) (minutes) (min-max)	14 (10-26)	14 (9-35)	.84^a^

^a^Mann–Whitney *U* test; ^b^Column percentage; ^c^Pearson Chi-square test; ^d^Fisher’s exact test BMI, body mass index.

**Table 4.** VAS Scores of the Patients

VAS—30^th^ Minute	n (%)
No symptoms	212 (91.8)
Mild	18 (7.8)
Moderate	1 (0.4)
Severe	–
VAS—6^th^ hour	n (%)
No symptoms	218 (94.4)
Mild	13 (5.6)
Moderate	–
Severe	–
VAS—24^th^ hour	n (%)
No symptoms	228 (98.7)
Mild	3 (1.3)
Moderate	–
Severe	–

VAS, visual analog scale

**Table 5.** Comparison of VAS Scores for Left Lateral and Right Lateral Positions

	Left Lateral	Right Lateral	*P*
VAS—30^th^ minute n (%)			.26^d^
No symptoms	121 (93.8)	91 (89.2)	
Mild	8 (6,2)	10 (9.8)	
Moderate	–	1 (1)	
Severe	–		
Total	129 (100)	102 (100)	
VAS—6^th^ hour n (%)			.46^c^
No symptoms	123 (95.3)	95 (93.1)	
Mild	6 (4.7)	7 (6.9)	
Moderate	–	–	
Severe	–	–	
Total	129 (100)	102 (100)	
VAS—24^th^ hour n (%)			.58^d^
No sypmtoms	128 (99.2)	100 (98)	
Mild	1 (0.8)	2 (2)	
Moderate	–	–	
Severe	–	–	
Total	129 (100)	102 (100)	

^c^Pearson Chi-square test; ^d^Fisher’s exact test VAS, visual analog scale.

## Data Availability

The data that support the findings of this study are available on request from the corresponding author.
